# Diagnostic performance of ultrasound in the assessment of gastric contents: a meta-analysis and systematic review

**DOI:** 10.1186/s13244-024-01665-0

**Published:** 2024-03-27

**Authors:** Xuanyuan Pan, Jun Chai, Xin Gao, Si Li, Jie Liu, Linxing Li, Yanjing Li, Zhichao Li

**Affiliations:** 1 Department of Anesthesiology, Shengjing Hospital of China Medical University, No.36 Sanhao Street, Shenyang, Liaoning Province China; 2 Department of Anesthesiology, Cancer Hospital Chinese Academy of Medical Sciences, No.17 Panjiayuan Nanli, Beijing, Chaoyang District China

**Keywords:** Gastric contents, Meta-analysis, Reflux aspiration, Ultrasound

## Abstract

**Objective:**

To systematically analyze the accuracy of ultrasonic techniques in assessing the nature of gastric contents and their volume.

**Methods:**

English-language articles that used ultrasonic techniques to assess the nature of gastric contents and their volume in patients were selected. In eligible studies, data were recalculated and analyzed for forest plots and subject summary curves of operating characteristics (SROC). Study quality was assessed using the diagnostic accuracy study quality assessment tool QUADAS-2. Publication bias was tested using funnel plots.

**Results:**

Nine articles with a total of 523 study subjects were identified for this review. All studies were feasibility studies. The sensitivity of ultrasound assessment of gastric contents ranged from 53 to 100% and the specificity from 48 to 99%. The combined analysis yielded an area under the working characteristic curve for subjects of 97% (95% confidence interval (CI), 95–98%), a sensitivity of 95% (95% CI, 84–99%), and a specificity of 88% (95% CI, 72–95%). There was a high degree of heterogeneity among the studies due to inter-operator differences and small sample sizes.

**Conclusion:**

Ultrasound techniques have high diagnostic accuracy in assessing the nature of gastric contents and their volume in patients. However, most of the studies were feasibility studies with small sample sizes, lacked standardization, and had high risk of bias. More studies are needed in the future to investigate the diagnostic performance of gastric ultrasound assessment techniques.

**Critical relevance statement:**

Ultrasonography can be used to assess gastric contents, but standardized data integration and reporting are needed to account for the diagnostic capabilities of this technology.

**Key points:**

• Ultrasound is a safe and feasible tool for assessing gastric contents.

• Ultrasound has good diagnostic performance for gastric contents.

• There is still a certain heterogeneity within our analysis process; more research is needed in the future to improve our results.

**Graphical Abstract:**

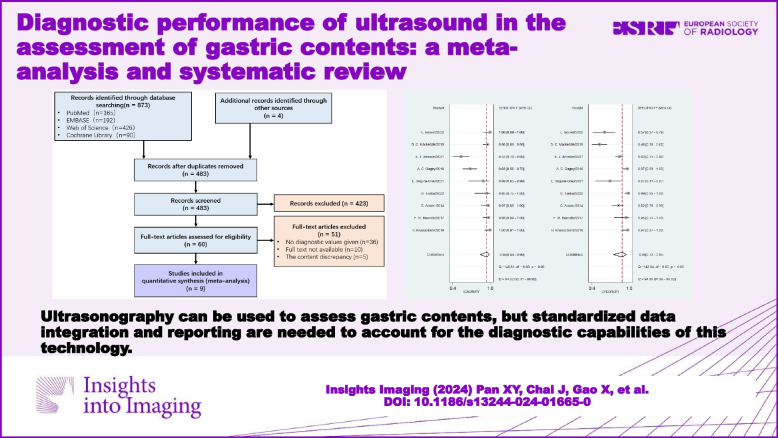

**Supplementary Information:**

The online version contains supplementary material available at 10.1186/s13244-024-01665-0.

## Background

Reflux aspiration of gastric contents is one of the major perioperative complications and can lead to permanent and severe damage or even death, such as aspiration pneumonia, acute respiratory distress syndrome, multi-organ dysfunction, and brain damage. A research survey found that incidents related to aspiration pneumonia accounted for 5–9% of anesthesia-related adverse events during the perioperative period, while the mortality rate for patients who experienced aspiration was as high as 10–60% [1–3]. The occurrence and prognosis of aspiration pneumonia are mainly related to the status of gastric contents and pH. The more residual gastric contents present, the lower the pH value, and the presence of solid residual contents portends greater risk of aspiration pneumonia, resulting in more severe complications. Therefore, preoperative assessment of the nature and volume of gastric contents is of great significance in preventing perioperative gastric content reflux and pulmonary aspiration [4–6]. In addition, a reasonable duration of preoperative fasting is one of the important components of the enhanced recovery after surgery (ERAS) strategy, and excessive or insufficient fasting is detrimental to the patient’s early postoperative recovery. The refinement and development of preoperative fasting protocols also require an accurate assessment of gastric contents in patients of different ages and states [7].

Currently, clinical methods used to measure gastric contents include upper gastrointestinal imaging, three-dimensional CT, MRI, and other imaging techniques. However, most of these techniques cannot be applied to patients with gastrointestinal dynamics disorders, such as intestinal obstruction and gastric antrum occupation. In addition, acetaminophen absorption, gastric impedance, scintigraphy, or polyglycol dilution can also be used to indirectly determine the gastric contents, or gastric microscopy, direct gastric aspiration, and gastric blind aspiration allow for direct measurement of gastric contents, but these procedures are invasive and can cause damage to the patient in addition to being poorly tolerated [8–10].

Gastrointestinal ultrasonography has been proposed since the early 1980s for the diagnosis of gastrointestinal pathology, examination of gastrointestinal kinetics, and to study factors affecting gastric emptying [11–13]. Ultrasonography was first utilized in 1992 for the detection of gastric contents [14], and in 1993, Fujigaki et al. proposed to indirectly calculate the volume of gastric contents through the measurement of the “cross-sectional area of gastric antrum (CSA),” thereby providing a more accurate, quantitative, assessment of gastric contents [15]. In recent years, ultrasound techniques have been used for gastric contents assessment mainly including their nature and volume. The main methods that have been proposed are quantitative assessment, qualitative assessment, and graded assessment [16]. Ultrasonography is convenient and noninvasive and has few contraindications, making it a tool that can be rapidly utilized at the bedside [17]. Current studies generally agree that ultrasound has the potential to be a safe and feasible tool for the assessment of intragastric material in daily clinical practice [16, 18].

The available literature summarizes the characteristics of ultrasonography of gastric contents and describes the existing assessment methods, but there is no systematic review of the diagnostic performance of ultrasound for this purpose and the diagnostic performance of ultrasound for the “high-risk stomach” has not yet been conclusively determined [19, 20]. This meta-analysis and systematic review summarizes the current diagnostic performance and limitations of ultrasound techniques for assessing the nature and volume of gastric contents, and points out directions for future applications in this field.

## Materials and methods

### Search strategy

This system evaluation and meta-analysis follow the Preferred Reporting Items for Systematic Reviews and Meta-Analyses Diagnostic Test Accuracy (PRISMA-DTA) statement [21] (Supplementary File 1). We systematically reviewed all studies that reported data on the accuracy of the assessment of the nature of gastric contents and their volume using ultrasound techniques. Original articles published in English from January 1, 2000, to June 23, 2023, were searched in PubMed, EMBASE, Web of Science, and the Cochrane Library using "ultrasound", "gastric contents", and "assess" as search terms, and the complete search strategy is shown in the Supplementary File 2. Manually retrieve original research and review papers and references cited in the papers and cross-reference them to ensure that all relevant literature is found. We registered the systematic review protocol on PROSPERO (CRD42023448022).

### Inclusion and exclusion criteria

Studies meeting the following inclusion criteria were selected: (1) study and design used ultrasound as one of the methods to evaluate gastric contents; (2) results of observation of gastric contents under ultrasound were described and analyzed; (3) literature that extrapolates or gives direct data on the number of true positives, false positives, true negatives, and false negatives in the 2 × 2 list. Studies meeting the following exclusion criteria were rejected: (1) exclude reviews, case reports, animal experiments, conference abstracts, and other types of articles; (2) duplicated data, different articles published by the same author were excluded from the use of duplicated data by reading the full text. Two researchers (X.Y.P. and J.L.) conducted a literature search, screened titles and abstracts, and selected appropriate studies for full-text review based on predefined inclusion and exclusion criteria. Any disagreements were resolved through discussion with a third senior researcher (J.C.).

### Data extraction

Data extraction was carried out independently by 2 researchers (X.G. and S.L.), and in case of controversy during the extraction process, a third researcher (J.C.) determined the final information to be used. The contents included in the literature extraction include author, publication time, publication country, sample number, research design, research object, gold standard, diagnostic threshold, ultrasound operator, patient’s posture, observation site, measurement plane, evaluation method of gastric contents (grading/quantitative/qualitative), true positive (TP), false negative (FN), false positive (FP), true negative (TN), and 2 × 2 tables that were computed. If the study did not report the original diagnostic data, we reconstructed 2 × 2 tables based on the diagnostic estimates provided in the article. For the literature on ultrasonic examination by several operators, the data of different operators were included in the analysis. When data were not explicit or missing, an investigator (L.X.L.) contacted the authors to obtain data. The study was excluded when no response was received (at least three attempts).

### Quality assessment

The quality of study reports was assessed by us using a modified version of the Quality Assessment Tool for Diagnostic Studies (QUADAS-2) [22]. The Quadras-2 tool mainly consists of four parts: patient selection, index test, reference standard, and patient flow and timing. All components will be evaluated in terms of bias risk, and the first three components will also be evaluated in terms of clinical applicability. Quality assessment was performed independently by 2 investigators (Y.J.L. and X.Y.P.), and in case of any discrepancies, they were resolved through consultation with a third researcher (Z.C.L).

### Diagnostic performance and heterogeneity

We extracted a 2 × 2 list from all eligible studies, including TP, FN, FP, and TN. The presence of a full stomach or a “high-risk stomach” is considered positive. Then, the analysis data were summarized by Stata MP14(64-bit), and the sensitivity, specificity, and their corresponding 95% confidence intervals (95% CI) were combined by MIDAS. We use a random effect model to estimate the results. In addition, we also drew the receiver’s work characteristic curve (SROC). We calculated the area under SROC (AUC) to measure the diagnostic efficiency of gastric ultrasound. AUC close to 1 indicates good diagnostic performance. To quantify the statistical heterogeneity among the pooled studies, the Cochran-Q test and *I*^2^ index of diagnostic odds ratio were used. When the Cochran-Q test was *p* < 0.01, the heterogeneity among the included studies was statistically significant, and when the *I*^2^ index exceeded 50%, the heterogeneity was considered more obvious. We used MetaDiSc 1.4 software to test the threshold effect by the Spearman correlation coefficient between sensitivity and (1 − specificity), and the *p*-value of the Spearman correlation coefficient is greater than 0.05, which indicated that there was no threshold effect among the studies. When there was heterogeneity caused by non-threshold effect between included studies, meta-regression analysis was used to explore the possible sources of heterogeneity in the study with the following covariables as predictive variables: ultrasound operators (clinicians or ultrasound doctors), study types (RCT or cohort studies), patient types (healthy volunteers or special patients), publication time of articles (whether published within 5 years), positive diagnostic criteria (qualitative evaluation or quantitative evaluation), and further.

## Results

### Literature search

**Fig. 1:**
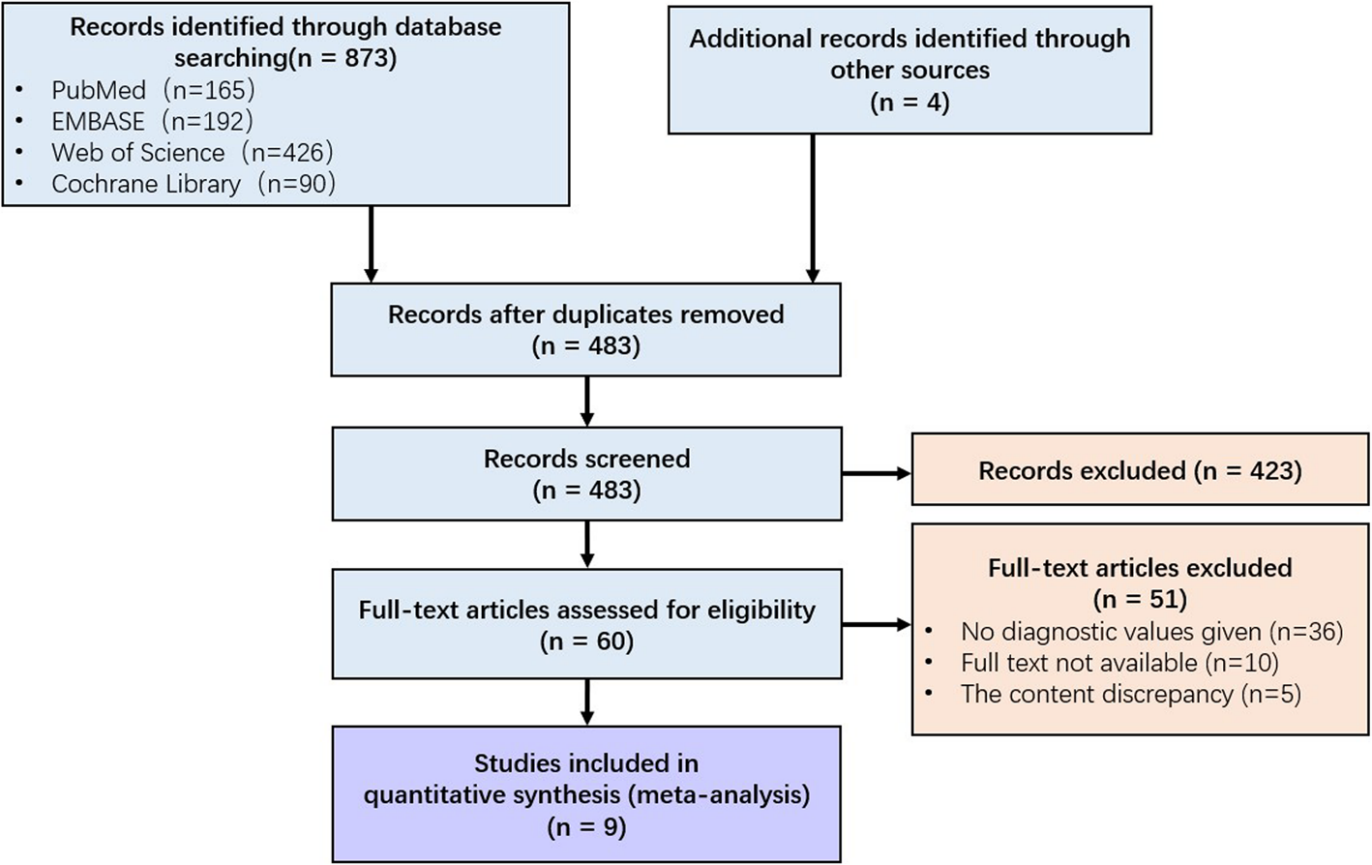
Flow diagram of the study selection process for the systematic review and meta-analysis

The initial search of the database retrieved a total of 877 articles, and after removing duplicates, a total of 483 studies were retained. Four hundred and twenty-three articles were excluded by screening titles and abstracts. After evaluation of the full text of 60 papers, nine studies met the inclusion criteria [4, 23–30]. The included studies cumulatively evaluated 523 subjects. Figure 1 shows the flow chart of the identification, screening, eligibility, and selection process using PRISMA guidelines.

### Study characteristics

The nine articles included four randomized controlled studies [4, 28–30] and five cohort studies [23–27]. Seven articles used randomization to fluid intake (clarified liquids/milk), solid food, or fasting in subgroups as the gold standard for assessing the accuracy of gastric ultrasound, and six of these studies were conducted in healthy volunteers [4, 23, 25, 28–30], whereas the study by Arzola et al. was conducted in women with late-term pregnancies [24]. Segura-Grau et al. studied patients requiring upper gastrointestinal endoscopy, using endoscopic findings of gastric contents as the gold standard [26]. The study by Gagey et al. was conducted in children, after anesthesia induction and tracheal intubation, the researchers recorded the nature and volume of stomach contents after being sucked out through a nasogastric tube, which was regarded as the gold standard [27].

In three studies, solid gastric content/mucous or clarified liquid > 1.5 mL/kg was defined as a “positive” diagnostic test—a “high-risk stomach” with gastric content exceeding the safe threshold for reflux aspiration. “Negative” was defined as no solid stomach contents or clear liquid in the supine position (SP) or right lateral position (RLD) < 1.5 mL/kg [4, 23, 30]. In one study, the positive diagnostic threshold was the presence of clear liquid > 0.8 mL.kg/L, viscous fluid, or solid stomach contents [27]. However, the positive threshold of diagnosis was not clearly defined in five articles. For these studies, we defined the intake of solid food as positive and the intake of liquid food as negative based on subgroups [24–29]. The study characteristics and results of the eligible articles are shown in Table 1.

**Table 1:** Characteristics of included articles and findings

Author	Year	country	Sample size	Mean age (years)	Sex (M/F)	Research design		Study object	Reference test	Positivity threshold	Ultrasound operator	Position	Area	Plane	Assessment methodology
Kruisselbrink et al [4]	2019	Canada	40	37	19/21		RCT	Adult	RG	Solid or liquid > 1.5 mL/kg	Clinician	SP RLDP	Antrum	Sagittal plane	Quantitatively Qualitatively
Bisinotto et al [23]	2017	France	67	NA	23/44		Cohort study	Adult	RG	Solid or liquid > 1.5 mL/kg	Sonologist	SP RLDP	Antrum	Sagittal plane	Qualitatively Quantitatively
Arzola et al [24]	2014	England	96	32	0/96		Cohort study	Pregnant	RG	NA	Clinician	SP RLDP	Antrum	Sagittal plane	Qualitatively
Tankul et al [25]	2022	Thailand	47	42	15/32		Cohort study	Adult	RG	NA	Clinician	RLDP	Antrum	Sagittal plane	Qualitatively Quantitatively
Segura-Grau et al [26]	2021	Spain	36	43.78	20/16		Cohort study	Adult	UGI	NA	Sonologist Clinician	SP RLDP	Antrum	Sagittal plane	Qualitatively Quantitatively
Gagey et al [27]	2018	France	130	NA	NA		Cohort study	Children	Aspiration	Solid or liquid > 0.8 mL/kg	Sonologist	SP RLDP	Antrum	Sagittal plane	Grade
Johnson et al [28]	2021	America	42	27	NA		RCT	Adult	RG	NA	Sonologist Clinician	SP RLDP	Antrum	Sagittal plane	Qualitatively
Mackenzie et al [29]	2019	America	45	32	30/15		RCT	Adult	RG	NA	Sonologist	SP RLDP	Antrum	NR	Qualitatively
Bouvet et al [30]	2022	France	20	27	9/11		RCT	Adult	RG	Solid or liquid > 1.5 mL/kg	Clinician	SP RLDP	Antrum	Sagittal plane	Grade quantitatively

*NA* not available, *RCT* randomized clinical trial, *RG* randomized grouping, *UGI* upper gastrointestinal endoscopy, *SP* supine position, *RLDP* right lateral position

### Quality assessment

**Fig. 2:**
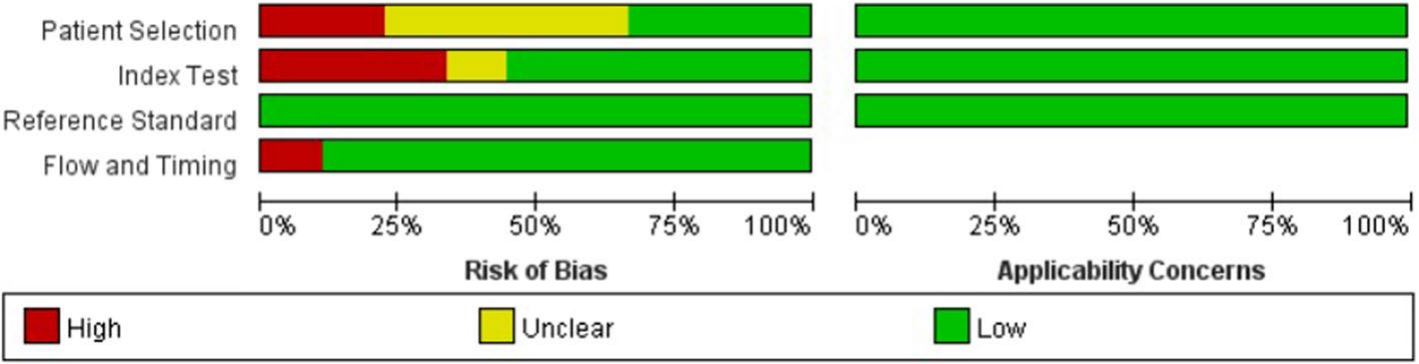
Quality Assessment of Diagnostic Accuracy Studies-2 (QUADAS-2) criteria for the included studies

The results of the quality assessment using QUADAS-2 are shown in Fig. 2. Overall, a high risk of bias was identified in the majority of studies, and in terms of case selection, 2 articles had case controls in the study design and did not explicitly describe the inclusion of cases as consecutive or randomized, resulting in a high risk of bias (22%) [28, 29]. Regarding the trials to be evaluated, three articles were at high risk (40%) [24, 25, 28], mainly because the threshold for diagnosing “high-risk stomach” was not given in these articles. There was an article that excluded patients with uncertain qualitative ultrasound evaluation and did not analyze all the included subjects, so there was a high risk in the case progress process [27]. No obvious problems were found in the reference experiment, and no obvious risks were found in clinical applicability (Supplementary File 3).

### Diagnostic performance

**Fig. 3:**
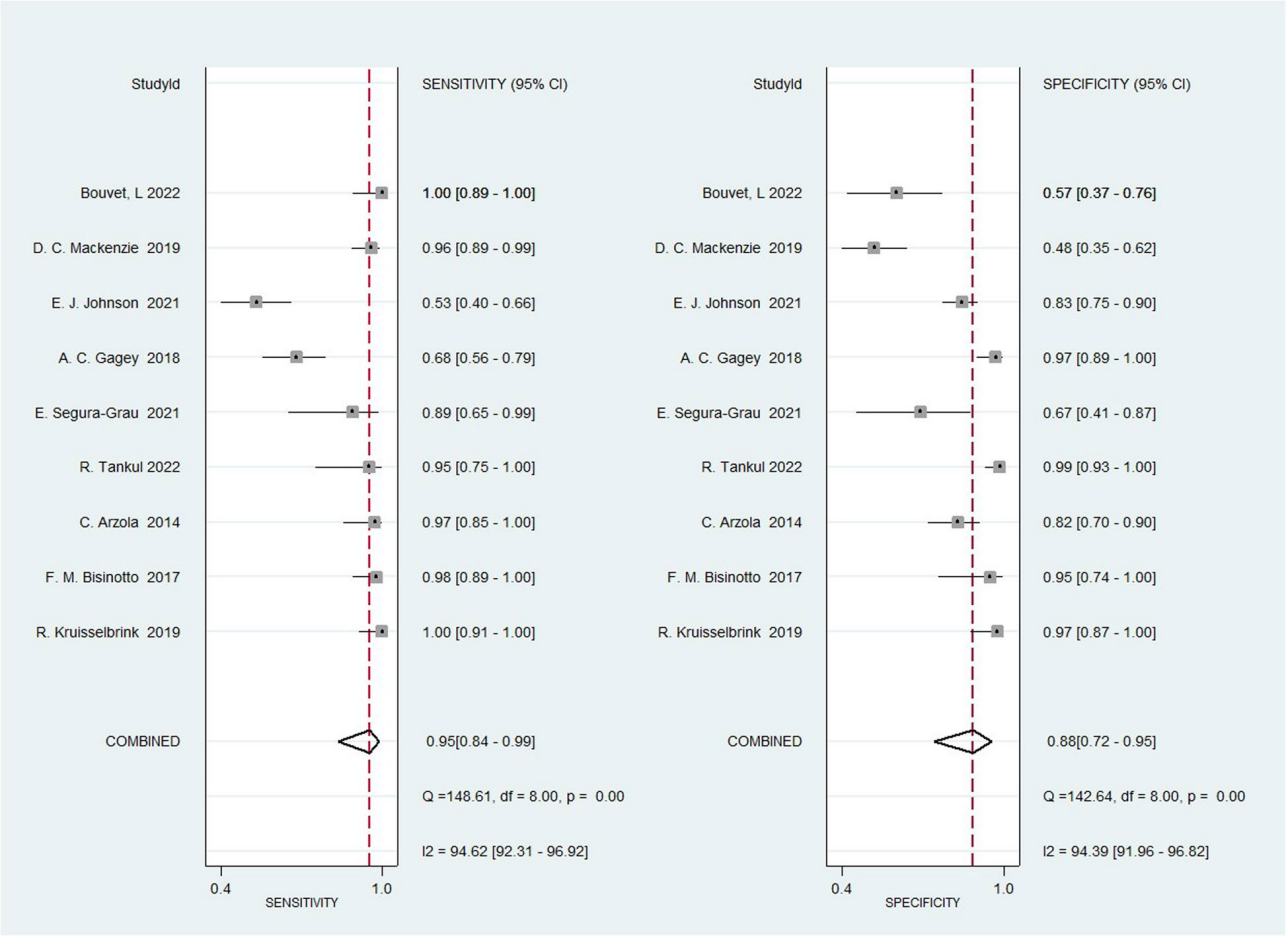
Combined forest plots for the sensitivity and specificity of ultrasound evaluation of gastric contents

**Fig. 4:**
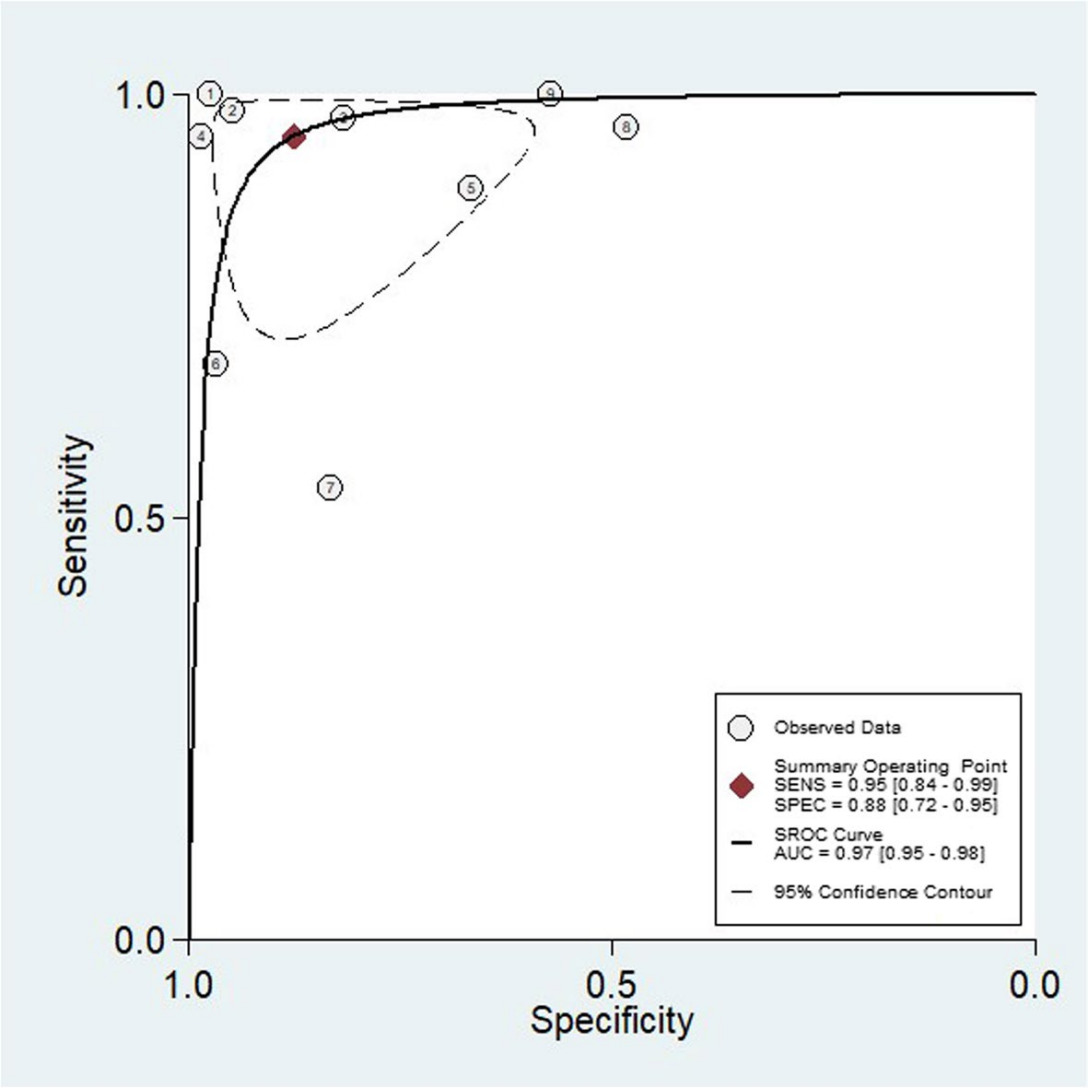
The summary receiver operating characteristic curve (SROC) for ultrasound evaluation of gastric contents

The sensitivity of the studies ranged from 53 to 100% and the specificity ranged from 48 to 99%. Analysis of all subjects combined yielded an area under the SROC curve of 97% (95% CI, 95–98%), sensitivity of 95% (95% CI, 84–99%), and specificity of 88% (95%CI, 72–95%) (Figs. 3 and 4). Analysis of the data yielded a non-significant Spearman correlation coefficient of 0.150 (*p* = 0.700 > 0.05) between the logarithm of sensitivity and the logarithm of specificity for gastric ultrasonography, and the SROC curves were not plotted with a “shoulder-arm shape,” implying that there was no threshold effect in this study. The combined sensitivity* I*^2^ index was 94.62% and the specificity *I*^2^ index was 94.39%, which corresponded to a high degree of statistical heterogeneity, and the Cochran-Q test of the diagnostic odds ratio yielded Cochran-Q = 44.84, *p* < 0.001, which implied that there was heterogeneity caused by non-threshold effects in this study.

### Meta‑regression analysis and subgroup analysis

The results of the meta-regression analysis showed a statistically significant effect of article publication time as well as positive diagnostic criteria on sensitivity (*p* < 0.05) (Table 2). According to the above covariate analysis, the heterogeneity has not been significantly improved. We can only conclude that different ultrasound examination operators and years of publication may have an impact on the heterogeneity, but it does not suggest that this variable is the ultimate cause of heterogeneity (Supplementary Figs. 1, 2), so the heterogeneity of this study cannot be effectively avoided. Finally, we use the random effect model to summarize the data.

**Table 2:** Results of bivariate meta-regression (sensitivity and specificity) and subgroup analysis

Parameter	Category	No	Se [95% CI]	*p*-value	Sp [95% CI]	*p*-value
Operator	Yes: sonologist	4	0.85 [0.68–1.00]	0.13	0.89 [0.75–1.00]	0.85
	No: clinician	5	0.97 [0.93–1.00]		0.84 [0.68–0.99]	
Design	Yes: RCT	4	0.94 [0.84–1.00]	0.77	0.77 [0.60–0.95]	0.05
	No: Cohort Study	5	0.94 [0.85–1.00]		0.93 [0.84–1.00]	
Patient	Yes: special patients	2	0.89 [0.66–1.00]	0.91	0.92 [0.79–1.00]	0.33
	No: health volunteer	7	0.95 [0.88–1.00]		0.84 [0.71–0.96]	
Year	Yes: < 2018	2	0.98 [0.94–1.00]	*0.02	0.90 [0.71–1.00]	0.50
	No: ≥ 2018	7	0.91 [0.83–1.00]		0.85 [0.73–0.97]	
Criteria	Yes: quantitative	4	0.98 [0.93–1.00]	*0.04	0.93 [0.83–1.00]	0.36
	No: qualitative	5	0.90 [0.78–1.00]		0.81 [0.65–0.96]	

^*^p < 0.05, the difference was statistically significant. *95%CI* 95% confidence interval, *No* number of articles, *RCT* randomized controlled trial, *Se* sensitivity, *Sp* specificity

### Publication bias

**Fig. 5:**
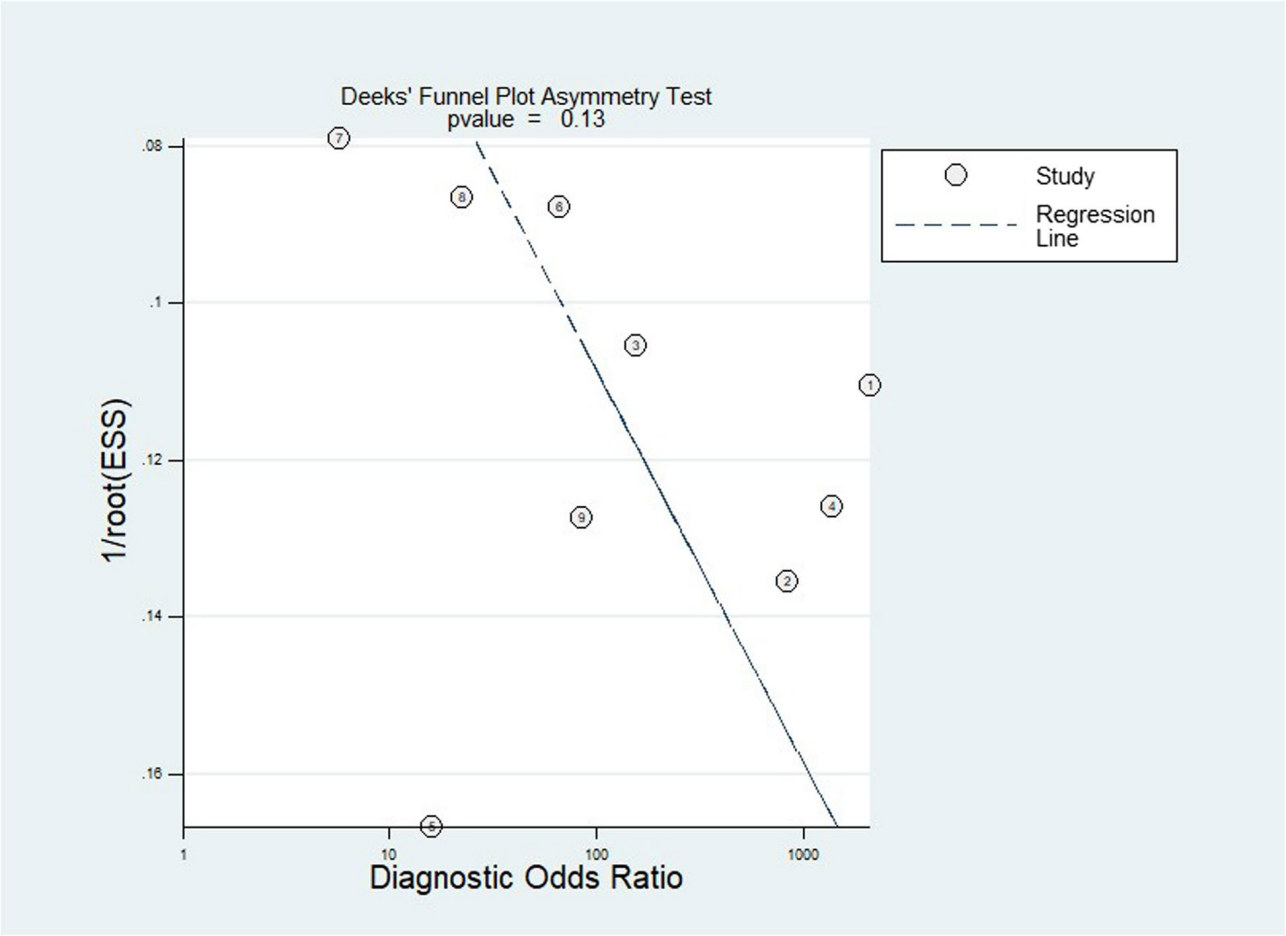
Deeks’ funnel plot used to evaluate potential publication bias

The Deek test showed *p* = 0.13 (> 0.05), indicating no publication bias in the included literature (Fig. 5).

## Discussion

This was the first systematic review and meta-analysis to use ultrasonography to assess the accuracy of gastric contents. The results showed that ultrasound had high diagnostic accuracy in assessing gastric contents, with an overall sensitivity of 95% and specificity of 88%, which suggests that ultrasound imaging has a good diagnostic performance for gastric content status and that a further combination of qualitative and quantitative assessment results can be effective in determining the risk of reflux aspiration. However, this result should be interpreted with caution due to the high degree of heterogeneity between studies and the high risk of bias found in most studies.

On the one hand, we performed a meta-regression analysis suggesting that the year of publication and the type of study had a significant effect on the sensitivity, which may be due to the continuous improvement of ultrasound equipment and the gradual standardization of related operations over time. Additionally, the study design process of the RCT avoided the inclusion of some patients who were difficult to diagnose or had unclear diagnostic results. This may have ultimately overestimated the diagnostic performance [31], consistent with the results of the meta-regression in which the diagnostic performance of the RCT group was slightly higher relative to the cohort study group. However, the differences in sensitivity and specificity between and within subgroups of the predictor variables were minor, and the overall results were stable. On the other hand, during the subgroup analysis, we also found that there was a high degree of heterogeneity in the clinician group compared with the ultrasound doctor group. Previous studies have shown that the measurement of gastric sinus cross-sectional area is highly reliable for sonographers with a lot of clinical experience, whether performed by the same operator or by different operators; whereas, although gastric ultrasonographic assessment is easy to learn, it should be performed before the operation, for both novice operators and non- sonographers. For less experienced operators, although gastric ultrasound assessment is easier to learn, a series of relevant trainings should be performed before the procedure [25, 32, 33]. Therefore, when performing gastric ultrasonography, the potential impact of differences in operator clinical experience should be carefully considered.

The gastric antrum is currently considered to be the most suitable site for the evaluation of gastric contents by ultrasound because it is the most receptive part of the stomach to ultrasound imaging due to its consistent shape, location, and air content. Additionally liquid or solid gastric contents flow to the antrum in supine and right lateral positions, which is more conducive to improving the accuracy of ultrasound testing [17]. Most of the studies used a low-frequency (2–5 Hz) ultrasound probe with ultrasound manipulation in the sagittal plane to assess the gastric contents in three ways: qualitative assessment, quantitative assessment, and grading system. The widely accepted clinical algorithm for further identifying the risk of reflux aspiration based on the assessment results is the one previously specified by Perlas et al. based on the results of the qualitative assessment and quantitative calculation of gastric contents; the presence of solid gastric contents or a volume of liquid gastric contents (VCG) > 1.5 mL/kg is considered to be a “high-risk stomach” at very high risk of reflux aspiration [34–36]. In addition, there have been a few studies defining the “high-risk stomach” using graded assessment results, as well as composite ultrasound scales combining graded assessment with a cross-sectional area of the gastric antrum [30, 37]. The results of the study of Bouvet et al. on these three calculation methods showed that, under the same position, the diagnostic performance of the three calculation methods is similar, with little difference in sensitivity, specificity, negative predictive value, and positive predictive value, and all of them were able to diagnose the “high-risk stomach” relatively accurately. In comparison, the graded assessment alone allows for quick conclusions without the need for calculations, whereas the composite ultrasound scale only requires the patient to be placed in the supine position for measurement of the cross-sectional area of the gastric antrum without the need for complex postural changes.

Accurate assessment of gastric contents can help guide the anesthesiologist in selecting a reasonable anesthetic induction and tracheal intubation protocol for patients at potential risk for reflux aspiration. The nature of the gastric contents and their volume can be assessed to determine the degree of risk for the patient to experience reflux aspiration. For example, in diabetic patients, patients with gastrointestinal obstruction, women with advanced pregnancy, or patients with stroke, routine abstinence from food and drink does not achieve a completely empty stomach due to the presence of physiologic or pathologic factors that cause impaired gastric emptying [24, 38]. Additionally, patients requiring emergency surgery are often at high risk of preoperative reflux aspiration because they are often unable to fast according to standard guidelines. For patients at low risk of reflux aspiration, a conventional induction protocol can be used; when patients are at high risk of reflux aspiration, anesthesiologists may consider postponing elective surgery, and for emergency surgery patients, methods such as placing a gastric tube for gastrointestinal decompression, performing rapid anesthesia induction, cricoid cartilage compression, and awake ciliopathic-guided tracheal intubation are used to achieve the goal of effectively preventing the occurrence of reflux aspiration [39–41]. The awake tracheal intubation technique preserves the patient’s pharyngeal reflexes, which can minimize the occurrence of reflux aspiration and ensure patient safety, but awake intubation increases patient tension and fear, and the invasive operation of intubation leads to airway trauma as well as significant increase in blood pressure and heart rate, which may induce cardiovascular and cerebrovascular complications in fragile patients such as those who are elderly, have a history of myocardial ischemia, or have a history of cerebrovascular disease [42, 43]. Accurate preoperative assessment of the status of gastric contents can effectively identify patients with empty stomachs and avoid discomfort caused by awake tracheal intubation or preoperative indwelling gastric tubes. Therefore, we believe that anesthesiologists can further optimize the anesthetic management plan by performing ultrasound gastric content assessment before anesthesia in patients with the potential risk of reflux aspiration based on a combination of patient fasting and abstinence from food and drink, type of surgery, medications, and past medical history [44].

With the feasibility of the gastric ultrasound evaluation method confirmed, it also provides a new and reliable research tool for the development of related fields. Drinking and fasting before the operation is one of the main means to control the gastric content. Traditional perioperative management suggests that fasting should be started at midnight before the operation to minimize the risk of aspiration. However, fasting from midnight will increase insulin resistance and discomfort in patients. Children or frail elderly people, diabetic patients, and emaciated patients with BMI < 19 kg/m^2^ may suffer adverse reactions such as hypovolemia, severe hypoglycemia, and hypotension, which is not conducive to early postoperative patients [43, 45]. Therefore, in recent years, more and more researchers have paid attention to enhanced recovery after surgery (ERAS) and take reduced preoperative fasting time and reasonable fasting as one of the important components of ERAS. Some studies have shown that the current implementation of routine preoperative fasting guidelines is not ideal. In clinical practice, due to the concern about the risk of reflux aspiration, children, obese patients, and other special populations have a longer fasting time than recommended, and the optimal fasting time for different types of patients is still controversial [7, 46, 47]. In recent years, some studies have introduced the method of ultrasonic evaluation of gastric content when discussing the factors affecting preoperative gastric emptying or the controversial issues in the current preoperative fasting program. For example, Bouvet et al. discussed for the first time whether chewing gum would increase the risk of reflux aspiration. Shin et al. studied the safety of preoperatively drinking carbohydrates in the elderly. All of them used the measurement results of gastric content under ultrasound as the basis for judging gastric emptying [48, 49]. The Practice Guide of Preoperative Fasting published by the American Society of Anesthesiologists Committee is constantly updated according to the conclusions of these latest studies [50]. Therefore, gastric ultrasound evaluation technology can provide a more reliable research tool for improving related research of preoperative fasting programs. By accurately evaluating the nature and capacity of gastric contents, we can judge the effectiveness of different fasting programs and promote the continuous development and perfection of ERAS programs.

There are some limitations in this study. First, there was significant heterogeneity among the included studies, which made it difficult to interpret the results of the meta-analysis. We think that the high heterogeneity in the analysis may come from the ongoing development of ultrasound technology, differences in operator experience, and the types of research, but were unable to make a more accurate analysis because of the limited information available. The number of articles in this study that can obtain complete diagnostic performance-related indicators for analysis was small, and we were not able to conduct a sensitivity analysis to reduce heterogeneity for studies with a high risk of bias. In this study, when analyzing five articles that did not clearly define the positive threshold of diagnosis, all of them were defined as negative according to the grouping situation because they could not get more accurate information about the stomach contents. We admit that this definition method is not accurate enough, because the subjects who ingest liquid may reach the diagnostic standard of “high-risk stomach” with the amount of liquid in the stomach > 1.5 mL/kg, and there is a risk of reflux aspiration, which may increase the false positive rate (FP) in our analysis and affect the accuracy of our results. Therefore, more research is needed in the future to discuss the accuracy of ultrasonic evaluation of gastric contents and provide more diagnostic data to further improve our analysis.

Secondly, most of the articles included in this analysis are healthy volunteers, including a study on children and a study on pregnant women, so the results of this study are more applicable to routine clinical subjects. In this study, we did not find that the two articles for these special populations had a significant impact on the final results, suggesting that our existing ultrasonic evaluation method of gastric contents also had certain applicability and good diagnostic performance in children and pregnant women. This was consistent with the conclusions of other related articles that were not included [51–53], and some scholars put forward a new prediction model for the nature and volume of gastric contents in pregnant women [54]. However, due to the complex differences between these special populations and healthy adults, more research is needed in the future to analyze the effectiveness of gastric ultrasound evaluation technology in special groups and more appropriate evaluation methods, to popularize this technology in a wider range.

## Conclusion

The results of this systematic review and meta-analysis show that ultrasound has high diagnostic accuracy in evaluating patients’ stomach contents. However, most of the existing studies on the determination of gastric contents by ultrasonic technology are feasibility studies with small sample sizes, lack standardization, and had high risk of bias. Therefore, more relevant research articles are needed to improve and expand the sample size. Ultrasonic evaluation of gastric contents is a convenient, feasible, and accurate imaging technology, which can be widely used in clinical work in the future.

### Supplementary Information

**Supplementary Material 1.**

## Data Availability

All data generated or analyzed during this study are included in this published article.

## Abbreviations

CI: Confidence intervals
FN: False negative
FP: False positive
PRISMA-DTA: Preferred Reporting Items for Systematic Reviews and Meta-Analyses of Diagnostic Test Accuracy Studies
QUADAS-2: Quality Assessment of Diagnostic Accuracy Studies-2
RCT: Randomized clinical trial
RLDP: Right lateral position
SP: Supine position
TN: True negative
TP: True positive
