# Supplementary material for: Diagnostic performance of ultrasound in the assessment of gastric contents: a meta-analysis and systematic review
Source: Insights Imaging. 2024 Mar 27;15:98. doi: 10.1186/s13244-024-01665-0 (PMC10973285; doi:10.1186/s13244-024-01665-0)
Supplement: Supplementary file 1 — Supplementary Material 1. [file 13244_2024_1665_MOESM1_ESM.pdf]

Diagnostic performance of ultrasound in the assessment of gastric contents: a meta-analysis and systematic review

ELECTRONIC SUPPLEMENTARY MATERIAL

Supplementary File 1: PRISMA-DTA Checklist and Abstracts Checklist

Section/topic	#	PRISMA-DTA for Abstracts Checklist item	Reported on page #
**TITLE and PURPOSE**			
Title	1	Identify the report as a systematic review (+/- meta-analysis) of diagnostic test accuracy (DTA) studies.	Title page
Objectives	2	Indicate the research question, including components such as participants, index test, and target conditions.	Page 1
**METHODS**			
Eligibility criteria	3	Include study characteristics used as criteria for eligibility.	Page 1
Information sources	4	List the key databases searched and the search dates.	Page 1
Risk of bias & applicability	5	Indicate the methods of assessing risk of bias and applicability.	Page 1
Synthesis of results	A1	Indicate the methods for the data synthesis.	Page 1
**RESULTS**			
Included studies	6	Indicate the number and type of included studies and the participants and relevant characteristics of the studies (including the reference standard).	Page 1
Synthesis of results	7	Include the results for the analysis of diagnostic accuracy, preferably indicating the number of studies and participants. Describe test accuracy including variability; if meta-analysis was done, include summary results and confidence intervals.	Page 1-2
**DISCUSSION**			
Strengths and limitations	9	Provide a brief summary of the strengths and limitations of the evidence	Page 2
Interpretation	10	Provide a general interpretation of the results and the important implications.	Page 2
**OTHER**			
Funding	11	Indicate the primary source of funding for the review.	Declarations section

Registration	12	Provide the registration number and the registry name	PROSPERO (CRD42023448022)

Adapted From: McInnes MDF, Moher D, Thombs BD, McGrath TA, Bossuyt PM, The PRISMA-DTA Group (2018). Preferred Reporting Items for a Systematic Review and Meta-analysis of Diagnostic Test Accuracy Studies: The PRISMA-DTA Statement. JAMA. 2018 Jan 23;319(4):388-396. doi: 10.1001/jama.2017.19163.

For more information, visit: **www.prisma-statement.org**.

Section/topic	#	PRISMA-DTA Checklist Item	Reported on page #
**TITLE / ABSTRACT**			
Title	1	Identify the report as a systematic review (+/- meta-analysis) of diagnostic test accuracy (DTA) studies.	Title page
Abstract	2	Abstract: See PRISMA-DTA for abstracts.	Page 1-2
**INTRODUCTION**			
Rationale	3	Describe the rationale for the review in the context of what is already known.	Page 3
Clinical role of index test	D1	State the scientific and clinical background, including the intended use and clinical role of the index test, and if applicable, the rationale for minimally acceptable test accuracy (or minimum difference in accuracy for comparative design).	Page 3-5
Objectives	4	Provide an explicit statement of question(s) being addressed in terms of participants, index test(s), and target condition(s).	Page 4-6
**METHODS**			
Protocol and registration	5	Indicate if a review protocol exists, if and where it can be accessed (e.g., Web address), and, if available, provide registration information including registration number.	PROSPERO (CRD42023448022)
Eligibility criteria	6	Specify study characteristics (participants, setting, index test(s), reference standard(s), target condition(s), and study design) and report characteristics (e.g., years considered, language, publication status) used as criteria for eligibility, giving rationale.	Page 7
Information sources	7	Describe all information sources (e.g., databases with dates of coverage, contact with study authors to identify additional studies) in the search and date last searched.	Page 6
Search	8	Present full search strategies for all electronic databases and other sources searched, including any limits used, such that they could be repeated.	Page 6
Study selection	9	State the process for selecting studies (i.e., screening, eligibility, included in systematic review, and, if applicable, included in the meta-analysis).	Page 6-7
Data collection process	10	Describe method of data extraction from reports (e.g., piloted forms, independently, in duplicate) and any processes for obtaining and confirming data from investigators.	Page 7-8
Definitions for data	11	Provide definitions used in data extraction and classifications of target condition(s), index test(s), reference	Page 7-8

extraction		standard(s) and other characteristics (e.g. study design, clinical setting).	
Risk of bias and applicability	12	Describe methods used for assessing risk of bias in individual studies and concerns regarding the applicability to the review question.	Page 8
Diagnostic accuracy measures	13	State the principal diagnostic accuracy measure(s) reported (e.g. sensitivity, specificity) and state the unit of assessment (e.g. per-patient, per-lesion).	Page 9-10
Synthesis of results	14	Describe methods of handling data, combining results of studies and describing variability between studies. This could include, but is not limited to: a) handling of multiple definitions of target condition. b) handling of multiple thresholds of test positivity, c) handling multiple index test readers, d) handling of indeterminate test results, e) grouping and comparing tests, f) handling of different reference standards	Page 7-8

Section/topic	#	PRISMA-DTA Checklist Item	Reported on page #
Meta-analysis	D2	Report the statistical methods used for meta-analyses, if performed.	Page 9-10
Additional analyses	16	Describe methods of additional analyses (e.g., sensitivity or subgroup analyses, meta-regression), if done, indicating which were pre-specified.	Page 9-10
**RESULTS**			
Study selection	17	Provide numbers of studies screened, assessed for eligibility, included in the review (and included in meta-analysis, if applicable) with reasons for exclusions at each stage, ideally with a flow diagram.	Page 10
Study characteristics	18	For each included study provide citations and present key characteristics including: a) participant characteristics (presentation, prior testing), b) clinical setting, c) study design, d) target condition definition, e) index test, f) reference standard, g) sample size, h) funding sources	Page 10-11 and Table 1
Risk of bias and applicability	19	Present evaluation of risk of bias and concerns regarding applicability for each study.	Page 13
Results of individual studies	20	For each analysis in each study (e.g. unique combination of index test, reference standard, and positivity threshold) report 2x2 data (TP, FP, FN, TN) with estimates of diagnostic accuracy and confidence intervals, ideally with a forest or receiver operator characteristic (ROC) plot.	Page 12-13
Synthesis of results	21	Describe test accuracy, including variability; if meta-analysis was done, include results and confidence intervals.	Page 12-13
Additional analysis	23	Give results of additional analyses, if done (e.g., sensitivity or subgroup analyses, meta-regression; analysis of index test: failure rates, proportion of inconclusive results, adverse events).	Page 13 and Table 2
**DISCUSSION**			
Summary of evidence	24	Summarize the main findings including the strength of evidence.	Page 14-21
Limitations	25	Discuss limitations from included studies (e.g. risk of bias and concerns regarding applicability) and from the review process (e.g. incomplete retrieval of identified research).	Page 21-23

Conclusions	26	Provide a general interpretation of the results in the context of other evidence. Discuss implications for future research and clinical practice (e.g. the intended use and clinical role of the index test).	Page 23
**FUNDING**			
Funding	27	For the systematic review, describe the sources of funding and other support and the role of the funders.	Page 23

Adapted From: McInnes MDF, Moher D, Thombs BD, McGrath TA, Bossuyt PM, The PRISMA-DTA Group (2018). Preferred Reporting Items for a Systematic Review and Meta-analysis of Diagnostic Test Accuracy Studies: The PRISMA-DTA Statement. JAMA. 2018 Jan 23;319(4):388-396. doi: 10.1001/jama.2017.19163.

For more information, visit: **www.prisma-statement.org**.

Supplementary File 2: Search strategy

Search strategy PubMed

((("Ultrasonography"[Mesh]) OR ((((((((((((((((((((((((Diagnostic Ultrasound[Title/Abstract]) OR (Diagnostic Ultrasounds[Title/Abstract])) OR (Ultrasound, Diagnostic[Title/Abstract])) OR (Ultrasounds, Diagnostic[Title/Abstract])) OR (Ultrasound Imaging[Title/Abstract])) OR (Imaging, Ultrasound[Title/Abstract])) OR (Imagings, Ultrasound[Title/Abstract])) OR (Echotomography[Title/Abstract])) OR (Ultrasonic Imaging[Title/Abstract])) OR (Imaging, Ultrasonic[Title/Abstract])) OR (Sonography, Medical[Title/Abstract])) OR (Medical Sonography[Title/Abstract])) OR (Ultrasonographic Imaging[Title/Abstract])) OR (Imaging, Ultrasonographic[Title/Abstract])) OR (Imagings, Ultrasonographic[Title/Abstract])) OR (Ultrasonographic Imagings[Title/Abstract])) OR (Echography[Title/Abstract])) OR (Diagnosis, Ultrasonic[Title/Abstract])) OR (Diagnoses, Ultrasonic[Title/Abstract])) OR (Ultrasonic Diagnoses[Title/Abstract])) OR (Ultrasonic Diagnosis[Title/Abstract])) OR (Echotomography, Computer[Title/Abstract])) OR (Computer Echotomography[Title/Abstract])) OR (Tomography, Ultrasonic[Title/Abstract])) OR (Ultrasonic Tomography[Title/Abstract])) OR (ultrasound[Title/Abstract]))) AND (("Gastrointestinal Contents"[Mesh]) OR (((((((((((Gastrointestinal Content[Title/Abstract]) OR (GI Contents[Title/Abstract])) OR (GI Content[Title/Abstract])) OR (Digestive Tract Contents[Title/Abstract])) OR (Digestive Tract Content[Title/Abstract])) OR (Stomach Contents[Title/Abstract])) OR (Stomach Content[Title/Abstract])) OR (Intestinal Contents[Title/Abstract])) OR (Intestinal Content[Title/Abstract])) OR (gastric contents[Title/Abstract])) OR (gastric content[Title/Abstract])))) AND (((assess[Title/Abstract]) OR (assessment[Title/Abstract])) OR (evaluate[Title/Abstract]))

Search strategy Embase

#1 ultrasound:ab,ti OR ultrasonography:ab,ti OR 'diagnostic ultrasound':ab,ti OR 'diagnostic ultrasounds':ab,ti OR 'ultrasound, diagnostic':ab,ti OR 'ultrasounds, diagnostic':ab,ti OR 'ultrasound imaging':ab,ti OR 'imaging, ultrasound':ab,ti OR 'imagings, ultrasound':ab,ti OR echotomography:ab,ti OR 'ultrasonic imaging':ab,ti OR 'imaging, ultrasonic':ab,ti OR 'sonography, medical':ab,ti OR 'medical sonography':ab,ti OR 'ultrasonographic imaging':ab,ti OR 'imaging, ultrasonographic':ab,ti OR 'imagings, ultrasonographic':ab,ti OR 'ultrasonographic imagings':ab,ti OR echography:ab,ti OR 'diagnosis, ultrasonic':ab,ti OR 'diagnoses, ultrasonic':ab,ti OR 'ultrasonic diagnoses':ab,ti OR 'ultrasonic diagnosis':ab,ti OR 'echotomography, computer':ab,ti OR 'computer echotomography':ab,ti OR 'tomography, ultrasonic':ab,ti OR 'ultrasonic tomography':ab,ti

#2 'gastric contents':ab,ti OR 'gastric content':ab,ti OR 'gastrointestinal contents':ab,ti OR 'gastrointestinal content':ab,ti OR 'gi contents':ab,ti OR 'gi content':ab,ti OR 'digestive tract contents':ab,ti OR 'digestive tract content':ab,ti OR 'stomach contents':ab,ti OR 'stomach content':ab,ti OR 'intestinal contents':ab,ti OR 'intestinal content':ab,ti

#3 assess:ab,ti OR assessment:ab,ti OR evaluate:ab,ti

#4 #1 AND #2 AND #3

Search strategy Web of Science (WoS)

#1 (TS=(Ultrasonography)) OR AB=(ultrasound or Diagnostic Ultrasound or Diagnostic Ultrasounds or Ultrasound, Diagnostic or Ultrasounds, Diagnostic or Ultrasound Imaging or Imaging, Ultrasound or Imagings, Ultrasound or Echotomography or Ultrasonic Imaging or Imaging, Ultrasonic or Sonography, Medical or Medical Sonography or Ultrasonographic Imaging or Imaging, Ultrasonographic or Imagings, Ultrasonographic or Ultrasonographic Imagings or Echography or Diagnosis, Ultrasonic or Diagnoses, Ultrasonic or Ultrasonic Diagnoses or Ultrasonic Diagnosis or Echotomography, Computer or Computer Echotomography or Tomography, Ultrasonic or Ultrasonic Tomography) and Preprint Citation Index

#2 (TS=(Gastrointestinal Contents)) OR AB=(gastric contents or gastric content or Gastrointestinal Content or GI Contents or GI Content or Digestive Tract Contents or Digestive Tract Content or Stomach Contents or Stomach Content or Intestinal Contents or Intestinal Content) and Preprint Citation Index

#3 AB=(assess or assessment or evaluate) and Preprint Citation Index

#4 #1 AND #2 AND #3 and Preprint Citation Index

Search strategy Cochrane

#1 (ultrasound):ti,ab,kw

#2 (Ultrasonic Diagnosis):ti,ab,kw OR (Echotomography, Computer):ti,ab,kw OR (Computer Echotomography):ti,ab,kw OR (Tomography, Ultrasonic):ti,ab,kw OR (Tomography, Ultrasonic):ti,ab,kw

#3 (Ultrasonographic Imagings):ti,ab,kw OR (Echography):ti,ab,kw OR (Diagnosis, Ultrasonic):ti,ab,kw OR (Diagnoses, Ultrasonic):ti,ab,kw OR (Ultrasonic Diagnoses):ti,ab,kw

#4 (Sonography, Medical):ti,ab,kw OR (Medical Sonography):ti,ab,kw OR (Ultrasonographic Imaging):ti,ab,kw OR (Imaging, Ultrasonographic):ti,ab,kw OR (Imagings, Ultrasonographic):ti,ab,kw

#5 (Imaging, Ultrasound):ti,ab,kw OR (Imagings, Ultrasound):ti,ab,kw OR (Echotomography):ti,ab,kw OR (Ultrasonic Imaging):ti,ab,kw AND (Imaging, Ultrasonic):ti,ab,kw

#6 (Diagnostic Ultrasound):ti,ab,kw OR (Diagnostic Ultrasounds):ti,ab,kw OR (Ultrasound, Diagnostic):ti,ab,kw OR (Ultrasounds, Diagnostic):ti,ab,kw OR (Ultrasound Imaging)

#7 MeSH descriptor: [Ultrasonography] explode all trees

#8 #7 OR #6 OR #5 OR #4 OR #3 OR #2 OR #1

#9 MeSH descriptor: [Gastrointestinal Contents] explode all trees

#10 (Gastrointestinal Content):ti,ab,kw OR (GI Contents):ti,ab,kw OR (GI Content):ti,ab,kw OR (Digestive Tract Contents):ti,ab,kw OR (Digestive Tract Content):ti,ab,kw

#11 (Stomach Contents):ti,ab,kw OR (Stomach Content):ti,ab,kw OR (Intestinal Contents):ti,ab,kw OR (Intestinal Content):ti,ab,kw

#12 (gastric contents):ti,ab,kw OR (gastric content):ti,ab,kw
#13 #9 OR #10 OR #11 OR #12
#14 (assess):ti,ab,kw OR (assessment):ti,ab,kw OR (evaluate):ti,ab,kw 634861
#15 #8 AND #13 AND #14

Supplementary File 3: Risk of bias assessment adapted from QUADAS-2

Adapted from *Whiting PF, Rutjes AW, Westwood ME, Mallett S, Deeks JJ, Reitsma JB, et al. QUADAS-2: a revised tool for the quality assessment of diagnostic accuracy studies. Ann Intern Med 2011; 155(8):529-536.*

<u>Domain 1: Patient Selection</u>

1.1 Risk of Bias: Could the selection of patients have introduced bias?
Signaling questions and answer guidelines
Signaling question 1: Was a consecutive or random sample of patients or specimens enrolled?

- Yes: the study enrolled a consecutive or random sample of eligible patients
- No: the study selected patients by selection or convenience
- Unclear: the study did not report how the patient selection was

Signaling question 2: Was a case-control design avoided?

- Yes: the study is not a case-control design
- No: the study is a case-control design
- Unclear: the study design was not reported, or we were unable to identify from the text

Signaling question 3: Did the study avoid inappropriate exclusions?

- Yes: the study enrolled consecutive or random samples of eligible patients
- No: the study excluded samples based on their prior testing, as these exclusions significantly reduce the generalizability of a study's findings
- Unclear: the study did not report exclusion criteria, or we were unable to identify from the text

Risk of Bias was evaluated as 'low risk' if studies scored 'yes' on all the questions or two questions were answered with 'yes' and one with 'unclear'; 'high risk' if two or more questions were answered with 'no' or one question was answered with 'no' and two with 'unclear'; and 'unclear risk' if studies scored 'unclear' on all the questions, two questions are answered with 'unclear' and one with 'yes', two questions were answered with 'yes' and one with 'no', or each question was answered with 'yes', 'no' and 'unclear'.

1.2 Applicability: Are there concerns that the included patients and setting do not match the review question?

- Low concern: the study enrolled a broad study population in any setting
- High concern: the study inappropriately included only subjects with a high incidence of delayed gastric emptying
- Unclear concern: the population was not well characterized, or we could not identify if a study's patients did not match our review question
-

Domain 2: Index Test

2.1 Risk of Bias: Could the conduct or interpretation of the index test have introduced bias?

Signaling question 1: Were the index test results interpreted without knowing the reference standard results?

- Yes: the reference standard (randomized grouping/UGI/ aspiration) test results were blinded. The results of the ultrasound test were reported without the knowledge of the random group, or UGI/ stomach contents suction was performed after the ultrasound test
- No: results of reference standard were unblended.
- Unclear: we were unable to identify whether to blind, or did not know the specific blind process

Signaling question 2: If a threshold was used, was it pre-specified?

- Yes: the "high-risk stomach" threshold is based on studies that were established before the trial began
- No: the threshold of the "high-risk stomach" was personally selected to optimize sensitivity and specificity, leading to over-optimistic estimates of test performance or the threshold is not clearly defined
- Unclear: we could not determine whether the threshold for a "high-risk stomach" is predetermined

Risk of Bias was evaluated as 'low risk' if studies scored 'yes' on all the questions; 'high risk' if one or two questions were answered with 'no'; and 'unclear risk' if questions were answered with 'yes' and 'unclear'.

2.2 Applicability: Are there concerns that the index test, its conduct, or interpretation differ from the review question?

- Low concern: the procedure of gastric ultrasound and the interpretation of "high-risk stomach" is carried out according to the prescribed procedure
- High concern: the procedure of gastric ultrasound and the interpretation of "high-risk stomach" are inconsistent with previous studies or cannot clearly distinguish the state of gastric contents
- Unclear concern: the gastric ultrasound procedures or interpretation of results were not discussed in the study, or are we unsure how gastric ultrasound was performed or interpreted
-

Domain 3: Reference standard

3.1 Risk of Bias: Could the reference standard, its conduct, or its interpretation have introduced bias?

Signaling question 1: Is the reference standard likely to classify the target condition correctly? We will score "yes" for all studies

- Yes: the reference standard for interpretation of gastric ultrasound results was randomized grouping/UGI/ aspiration
- No: the reference standard for the interpretation of gastric ultrasound results were unreliable
- Unclear: the reference standard for the interpretation of gastric ultrasound results were insufficient, or we cannot identify them from the text

Signaling question 2: Were the reference standard results interpreted without knowing the index test results?

- Yes: studies where the randomized grouping/UGI/ aspiration result was interpreted blindly to the results of the gastric ultrasound
- No: studies where the randomized grouping/UGI/ aspiration result was not interpreted blindly to the results of the gastric ultrasound

- Unclear: we were unable to identify whether to interpret the reference results without knowing the results of the gastric ultrasound

3.2 Applicability: Are there concerns that the target condition defined by the reference standard does not match the question?

- Low concern: the setting of random groups, UGI and aspiration can clearly determine the nature and volume of stomach contents
- High concern: random grouping settings, UGI and aspiration could not clearly determine the nature and volume of stomach contents
- Unclear concern: were methods for determining the nature and volume of gastric contents not discussed in the study, or were we unable to determine how to proceed or interpret randomized Settings, UGI, and aspiration

<u>**Domain 4: Flow and timing**</u>

4.1 Risk of Bias: Could the patient flow have introduced bias?

Signaling question 1: Was there an appropriate interval between the index test and reference standard?

- Yes: in randomized trials, volunteers underwent gastric ultrasound immediately after food/water intake, or UGI and aspiration immediately after gastric ultrasound
- No: food/water intake or UGI and aspiration were not followed by gastric ultrasound
- Unclear: it was not discussed in the study, or we were unable to determine when gastric ultrasound and reference standards tests test were conducted or interpreted

Signaling question 2: Did all patients in the study receive the same reference standard?

- Yes: the study used the same reference criteria for the stomach contents of all subjects
- No: the study used different reference criteria for the stomach contents of all subjects
- Unclear: it was not defined in the study, or we were unable to interpret the used reference criteria

Signaling question 3: Were all patients included in the analysis?

- Yes: the whole population recruited into the study was included in the analysis, or any exclusion was adequately described
- No: participants were missing, or the study excluded samples without a given reason
- Unclear: not enough information was given to assess why participants were excluded from the analysis, or we were unable to find an explanation for the exclusion of samples

Risk of Bias was evaluated as 'low risk' if studies scored 'yes' on all the questions or two questions were answered with 'yes' and one with 'unclear'; 'high risk' if two or more questions were answered with 'no' or one question was answered with 'no' and two with 'unclear'; and 'unclear risk' if studies scored 'unclear' on all the questions, two questions are answered with 'unclear' and one with 'yes', two questions were answered with 'yes' and one with 'no', or each question was answered with 'yes', 'no' and 'unclear'

Summary of the quality assessment by using QUADAS-2

	Risk of bias				Concerns regarding applicability		
**Author (year)**	**Patient selection**	**Index test**	**Ref. standard**	**Flow and timing**	**Patient selection**	**Index test**	**Ref. standard**
Kruisselbrink et al (2019)	UC	L	L	L	L	L	L
Bisinotto et al (2017)	UC	L	L	L	L	L	L
Arzola et al (2014)	UC	H	L	L	L	L	L
Tankul et al (2022)	UC	H	L	L	L	L	L
Segura-Grau et al (2021)	L	UC	L	L	L	L	L
Gagey et al (2018)	L	L	L	H	L	L	L
Johnson et al (2021)	H	H	L	L	L	L	L
Mackenzie et al (2019)	H	L	L	L	L	L	L
Bouvet et al (2022)	L	L	L	L	L	L	L

Supplementary Figures

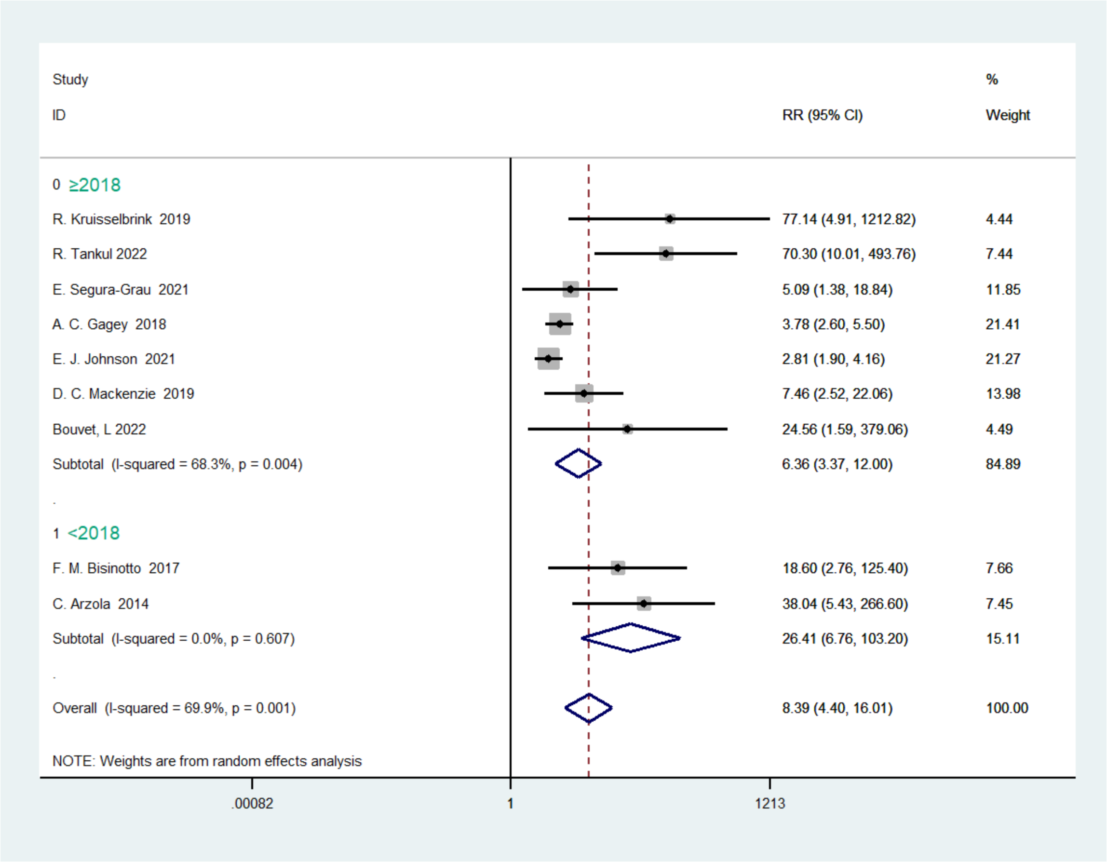

Supplementary Figure 1： Results of subgroup analysis based on year of study publication（I^2 <50%，P>0.05 indicates little intra-subgroup heterogeneity)

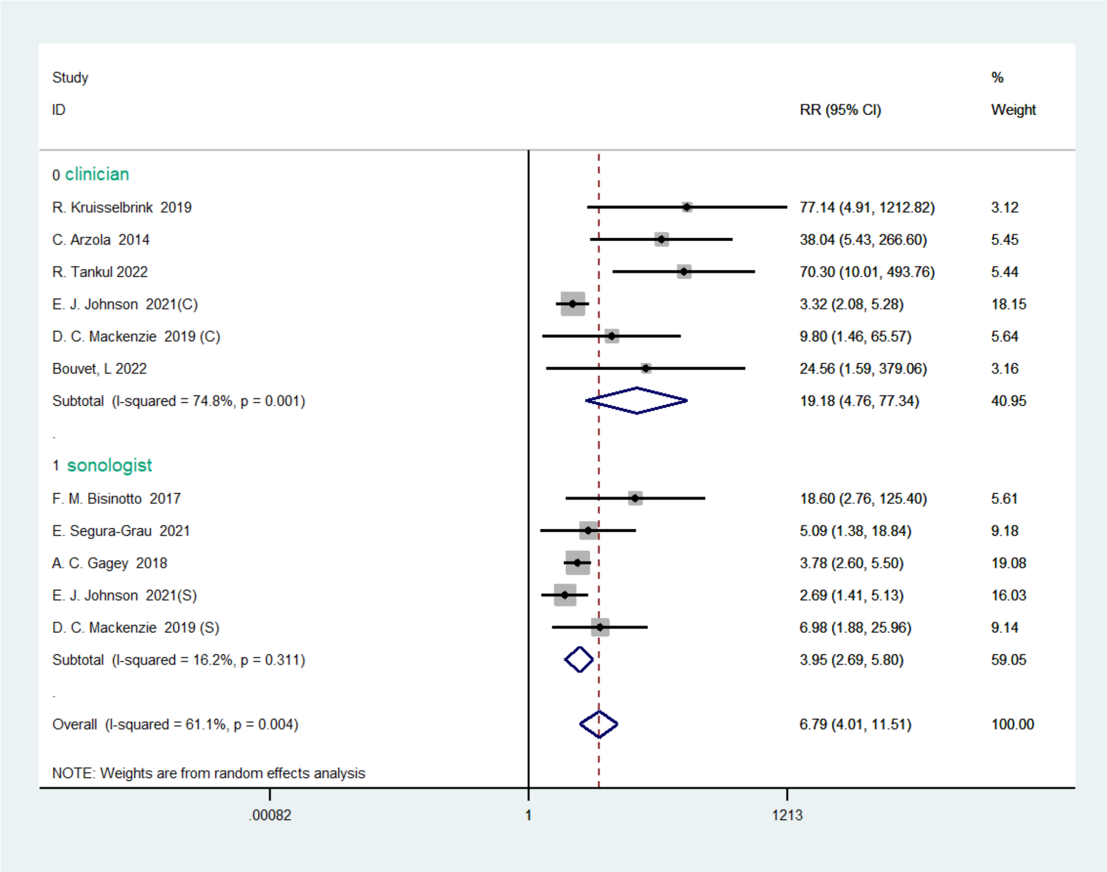

Supplementary Figure 2: Results based on analysis of subgroups of different ultrasound operators (sonographers or clinicians) （I^2 <50%，P>0.05 indicates little intra-subgroup heterogeneity)
